# Association between clinical symptoms and apolipoprotein A1 or apolipoprotein B levels is regulated by apolipoprotein E variant rs429358 in patients with chronic schizophrenia

**DOI:** 10.1186/s12991-021-00376-w

**Published:** 2021-12-20

**Authors:** Wenwang Rao, Xiangfei Meng, Keqing Li, Yunshu Zhang, Xiang Yang Zhang

**Affiliations:** 1 Department of Psychiatry, Faculty of Medicine and Health Sciences, McGill University, Montreal, QC Canada; 2 Mental Health and Society Division, Douglas Mental Health University Institute, Montreal, QC Canada; 3 Institute of Mental Health, Hebei Mental Health Centre, Hebei, China; 4 Department of Sleep Medicine, Hebei Psychiatric Hospital, Hebei, China; 5 CAS Key Laboratory of Mental Health, Institute of Psychology, Chinese Academy of Sciences, 16 Lin Cui Road, Beijing, 100101 China

**Keywords:** Apolipoprotein A1, Apolipoprotein B, Apolipoprotein E, Clinical symptoms, Schizophrenia

## Abstract

**Background:**

The apolipoprotein E (*ApoE*) gene polymorphisms are correlated with blood lipid levels and several neuropsychiatric symptoms. Therefore, this study aimed to examine whether the *ApoE* rs429358 affected the development and clinical symptoms of schizophrenia and to explore the relationship between apolipoproteins levels and clinical symptoms.

**Methods:**

The *ApoE* rs429358 was genotyped using a case–control design. The Positive and Negative Syndrome Scale (PANSS) was employed to evaluate the psychopathology of all patients.

**Results:**

A total of 637 patients with schizophrenia and 467 healthy controls were recruited. We found no significant differences in the genotype and allele distribution between the patient and control groups. A significant correlation between PANSS negative symptoms and ApoA1 levels (*p* = 0.048) or ApoB levels (*p* = 0.001) was found in patients with schizophrenia, which was also confirmed by linear regression analyses (*p* = 0.048 vs. *p* = 0.001). Interestingly, only in the T homozygote group, ApoA1 and ApoB levels were predictors of the PANSS negative symptom score (*p* = 0.008 vs. *p* = 0.012), while in the C allele carrier group, no correlation was observed.

**Conclusions:**

This study found that the levels of ApoA1 and ApoB were negatively associated with negative symptoms of patients with schizophrenia. Furthermore, the association between ApoA1 or ApoB levels and psychopathology of schizophrenia was regulated by *ApoE* rs429358.

## Introduction

Schizophrenia is a severe long-term mental disease, with an estimated lifetime prevalence of 0.4% ~ 0.88% [1–4], and may lead to high disability [5] and potential years of life loss [6]. A systematic review showed that the economic burden of schizophrenia was between 0.02 and 1.65% (that is, US$94 million–US$102 billion) of the gross domestic product (GDP) [7]. The Global burden of Disease 2016 (GBD 2016) study estimated that the burden of schizophrenia accounts for 1.7% of total years of life lived with disability (YLD) globally [8]. Studies have shown that there is a close relationship between changes in serum or membrane lipids and pathophysiology [9]. Patients with schizophrenia are characterized by psychiatric symptoms [10], and changes in blood lipid profiles [11, 12]. However, the biological mechanisms of underlying psychopathological symptoms and blood lipid profile changes in patients with schizophrenia remain unclear.

**Fig. 1:**
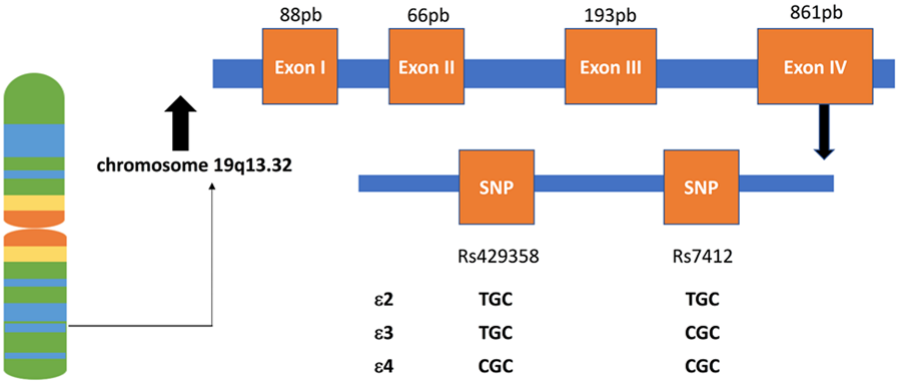
Schematic diagram of the human *ApoE* gene. The *ApoE* gene is located on chromosome 19 with four exons and two non-synonymous SNPs (rs429358 and rs7412) in exon 4 generate three major allelic variants (ε2, ε3, and ε4)

The Apolipoprotein E (*ApoE*) gene (4 exons and 3 introns) is located at 19q13.32, and has been described as a major candidate gene involved in neuropsychiatric genetics [13, 14]. It is well known that *ApoE* gene plays a key role in receptor-mediated endocytosis of lipoproteins in the brain [15] and affects downstream proteins, such as brain-derived neurotrophic factor (BDNF) [13, 16]. There are two functional polymorphism loci (rs429358 and rs7412) on exon 4 of *ApoE* gene, which combine to form three main isoforms (ε2, ε3, and ε4; Fig. 1) [17]. The minor allele (i.e., C) frequency of *ApoE* rs429358 from the Allele Frequency Aggregator (ALFA) project ranged from 0.026 in the East Asian population to 0.141 in the other African population [18]. The highest haplotype frequencies (rs440446- rs769449-rs769450-rs429358-rs7412) of *ApoE* gene in the 1000 Genomes were 0.292 (GGGCC) in the African population, 0.628 (CGGTC) in the Asian population, and 0.423 (GGATC) in the Europe population [19]. A previous systematic review and meta-analysis revealed the association between the *ApoE* ε3 and schizophrenia in Asian population [20]. In addition, there is evidence that *ApoE* ε4 is associated with a variety of neuropsychiatric symptoms (e.g., depression, anxiety, apathy, agitation, aggression, hallucinations, and delusions) in patient with Alzheimer’s disease [21–23]. Moreover, *ApoE* polymorphism may significantly affect lipid and lipoprotein levels [24]. In particular, one study found that *ApoE* rs429358 and *ApoE* rs7412 polymorphisms were significantly associated with low-density lipoproteins (LDL) levels in White and African Americans [25].

Clinical and epidemiological studies have found that changes in lipid profiles are also associated with a high risk of cardiovascular disease [26, 27], and schizophrenia is significantly associated with increased risk of cardiovascular diseases [28]. A previous study reported that changes in the blood lipid profiles were observed in patients with schizophrenia [29]. Apolipoprotein A1 (ApoA1) and apolipoprotein B (ApoB) are important components of apolipoproteins (Apos) and play a key role in lipid transport [30]. ApoA1 is the main protein component of high-density lipoproteins (HDL), and a biomarker of cardiovascular disease [31], while ApoB is a main protein in plasma chylomicrons, very-low-density lipoproteins (VLDL), and LDL [32]. The reference values of ApoA1 and ApoB levels in the adults (> 18 years) were ≥ 120 mg/dL and ≥ 100 mg/dL in males, and ≥ 140 mg/dL and ≥ 100 mg/dL in females [33]. The median ApoA1 and ApoB levels of health controls were 1.19 g/L and 0.86 g/L in the Africans, 1.15 g/L and 0.98 g/L in the Arabs, 1.23 g/L and 0.83 g/L in the Chinese, 1.26 g/L and 0.95 g/L in the Europeans, 1.18 g/L and 0.95 g/L in the Latin Americans, 1.07 g/L and 0.92 g/L in the South Asians, and 1.29 g/L and 0.96 g/L in the Southeast Asians, respectively [34]. A large number of studies have observed changes in the levels of ApoA1 and ApoB in patients with schizophrenia, with mixed results, such as elevated ApoA1 [31, 35, 36] or ApoB [37–39], which can be used as biomarkers of schizophrenia [39] as well as decreased ApoA1 [38, 40] or ApoB [36]. In addition, *ApoE* gene polymorphism may affect the expression levels of ApoA1 and ApoB [41–43]. For example, one study found that *ApoE* ε4 allele carriers had significantly higher levels of ApoB in the community cohort of older individuals [42], while another study found that *ApoE* ε2 allele carriers had significantly lower levels of ApoB in Chinese Han population [41]. Interestingly, the Alzheimer disease (AD) subjects carrying *ApoE* ɛ4 allele had lower ApoA1 levels but higher ApoB levels [44] and there were differences in biomarker levels (e.g., cholesterol, LDL, and ApoB) for each *ApoE* genotype with reference to ε3ε3 in a UK Biobank study [45].

As mentioned above, we speculated that *ApoE* polymorphism rs429358 may be involved in the development of schizophrenia and further the relationship between the clinical symptoms and the ApoA1 or ApoB levels in patients with schizophrenia. Therefore, the main purposes of this study were (1) to identify the association between *ApoE* polymorphism rs429358 and schizophrenia; (2) to analyze the relationship between ApoA1 or ApoB levels and clinical symptoms in patients with schizophrenia; and (3) to explore whether the relationship between serum levels of ApoA1 or ApoB and clinical symptoms was regulated by *ApoE* polymorphism rs429358.

## Materials and methods

### Study participants

Patients were recruited from Beijing Hui-Long-Guan hospital, and Hebei Rongjun Hospital in Baoding city near Beijing. They met the following inclusion criteria: (1) 18–70 years old; (2) diagnosed with schizophrenia by two psychiatrists using the Chinese version of Structured Clinical Interview for DSM-IV (SCID), with a Kappa value greater than 0.80; (3) having the diagnosis for at least 2 years to meet the eligibility of chronicity; (4) having taken a stable dose of oral antipsychotics for at least 12 months before entering this study; and (5) having no other psychiatric diagnoses.

In this study, healthy controls were randomly recruited through advertisements in the local community. They met the following inclusion criteria: (1) 18–70 years old; (2) no self-reported personal or family history of any type of psychiatric disorder; and (3) having not taken psychoactive drugs (e.g., antipsychotic, antianxiety, antidepressant, or mood-stabilizing drugs).

Participants were excluded if they met the following criteria: (1) diagnosed with drug or alcohol abuse/dependence; (2) suffering from major physical diseases, including head injury, epilepsy, cardiovascular disease, cerebrovascular disease, infection, cancer, and diabetes; and (3) pregnancy.

All participants voluntarily participated in this study and gave written informed consent. The protocol was approved by the Ethics Committee of Beijing Hui-Long-Guan hospital, and it was implemented in accordance with the Declaration of Helsinki [46].

### Data collection and measures

General information, socio-demographic characteristics, and medical conditions were collected by trained researchers from self-designed questionnaires and medical records. Four psychiatrists used the Positive and Negative Syndrome Scale (PANSS) to assess the clinical symptoms of patients [47, 48] on the same day as the whole blood sampling. These psychiatrists received a training in the use of PANSS before the start of this study. After training, they maintained an inter-rater correlation coefficient (ICC) greater than 0.8.

### DNA extraction and SNP genotyping

Genomic DNA was extracted from 5 ml of peripheral venous blood sample using the standard salting-out procedures [49], and then stored at -80 degrees. According to the protocol [50], *ApoE* polymorphism rs429358 was genotyped by using Matrix-Assisted Laser Desorption/Ionization Time of Flight Mass Spectrometry (MALDI-TOF–MS) (Sequenom Inc., San Diego, CA, USA). After querying the reference sequence of the NCBI GenBank database, primers and extension probes were generated. Re-genotyping was performed by trained researchers without knowing the clinical information in 5% randomly selected samples for quality control, with an error rate of less than 0.1%.

### Serum ApoA1 and ApoB levels measurement

Blood sampling and measurement of serum apolipoprotein (ApoA1 and ApoB) levels were described in detail in our previous studies [12, 51, 52], which were performed on the Olympus AU2700 analyzer using the immunoturbidimetric method (Beijing Leadman Biotechnology).

### Statistical analysis

The differences between cases and controls were measured by using independent two-sample t-test for continuous variables and Chi-squared (χ2) for categorical variables. The deviations from Hardy–Weinberg equilibrium (HWE) and genetic model (i.e., codominant, dominant, recessive, and over-dominant) analysis for *ApoE* rs429358 were examined using the SNPStats program, a web tool for SNP analysis (https://www.snpstats.net/start.htm) [53]. Further, in order to identify the effect of ApoE rs429358 on susceptibility to schizophrenia, logistic regression was used to control for confounding factors.

Since almost no homozygous variant CC genotypes were detected in our study (approximately 0.8% of the patients and 0.5% of healthy controls), we considered the CC and TC genotypes as a group in the following association analysis.

Since all demographic and clinical variables, as well as ApoA1 and ApoB levels were normally distributed in patients and healthy controls (Kolmogorov–Smirnov one sample test, all p > 0.05). An independent two-sample t-test was used to assess between-group differences grouped by genotypes for continuous variables, and Chi-squared (*χ*^2^) was used for dichotomous variables. Taking the *ApoE* rs429358 genotype as the independent variable and the PANSS total score and its subscale scores as the dependent variables, the analysis of covariance (ANCOVA) was further constructed, with those variables that showed significant differences in the above independent two-sample t-test or Chi-square test as covariates. Pearson correlation analysis was used to analyze the correlation between variables, and the partial correlation analysis was carried out with age, gender, education level, BMI, and age of onset as covariates. Linear regression analysis was used to explore whether there were differences in the relationships between ApoA1 or ApoB levels and PANSS scores among the *ApoE* rs429358 subgroups. The PANSS total or subscale scores were taken as dependent variables, and levels of ApoA1 and ApoB were used as independent variables for the whole sample and each *ApoE* rs429358 subgroup, adjusting for age, gender, education, BMI, and age of onset. Bonferroni corrections were applied to each test to adjust for multiple testing.

Power analysis was conducted using software Quanto (Version 1.2.3) under log additive, recessive, and dominant models, assuming that the prevalence rate of schizophrenia in the population was 1%. All data analyses were performed using SPSS, version 26.0 (IBM SPSS, IBM Corp., Armonk, NY, USA), with a significance level of 0.05 (two sided).

## Results

### Sample characteristics

**Fig. 2:**
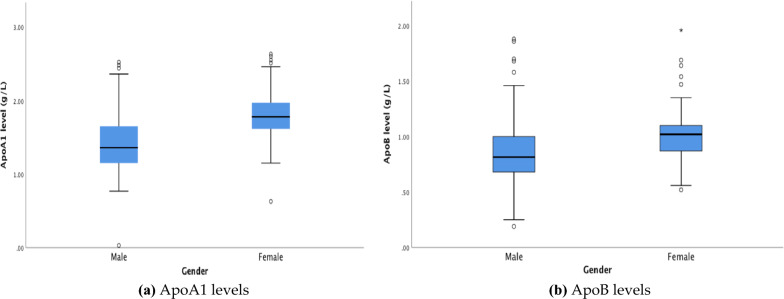
The ApoA1 (gl/L) and ApoB levels (gl/L) between the males and females

A total of 637 patients with schizophrenia and 467 healthy controls were included. Their demographic and clinical information are summarized in Table 1. Most patients received monotherapy with antipsychotic drugs, including clozapine (*n* = 245), risperidone (*n* = 116), chlorpromazine (*n* = 32), perphenazine (*n* = 26), sulpiride (*n* = 20), quetiapine (*n* = 19), aripiprazole (*n* = 14), olanzapine (*n* = 7), and haloperidol (*n* = 7). The average dose of antipsychotics (equivalent to chlorpromazine) was 391.72 ± 181.22 mg/day [54–56]. There were significant differences in gender, BMI, and age between patients and healthy controls (all p < 0.05), which were adjusted as covariates in the following analyses. In addition, there were sex differences in ApoA1 and ApoB levels (*t* = − 12.468, *p* < 0.001 vs. *t* = − 7.253, *p* < 0.001, Fig. 2) and no significant difference was found in ApoA1 and ApoB levels between typical antipsychotics and atypical antipsychotics (*t* = − 0.592, *p* = 0.554 vs. *t* = 1.181, *p* = 0.238). Besides, sex differences were found in PANSS positive symptoms (*t* = − 4.322, *p* < 0.001), PANSS negative symptoms (*t* = 5.598, *P* < 0.001), and PANSS general psychopathology (*t* = − 3.670, *p* < 0.001), except for PANSS total score (*t* = − 0.145, *p* = 0.885).

**Table 1:** Demographic profiles in patients with schizophrenia cases and controls (Mean ± SD)

Variables	Cases (n=637)	Controls (n=467)	t/X^2^	*p* value
Age (years)	47.52±10.61	44.94 ±13.63	− 3.392	0.001
Sex				
Female (%)	156 (24.5)	274 (58.7)	132.406	<0.001
Male (%)	481 (75.5)	193 (41.3)		
Years of education	9.30±6.81	9.66±5.33	0.955	0.340
Body mass index	24.45±4.00	25.14±4.18	2.538	0.011
Age of onset (years)	23.20±5.16	–	–	–
Duration of illness (years)	24.43 ± 10.52	–	–	–
Mean daily dose (mg/day) (chlorpromazine equivalents)	391.72 ± 181.22	–	–	–
PANSS total score	60.81 ± 14.91	–	–	–
Positive symptoms	11.90 ± 5.15	–	–	–
Negative symptoms	23.25 ± 8.50	–	–	–
General psychopathology	25.66 ± 6.02	–	–	–
ApoA1 level (g/L)	1.53 ± 0.38	–	–	–
ApoB level (g/L)	0.89 ± 0.24	–	–	–
ApoA1/ApoB	1.82 ± 0.62	–	–	–

### Association analysis of ApoE rs429358 with schizophrenia

The allele frequencies and genetic models are presented in Table 2. The HWE test revealed that the genotype distributions of the *ApoE* rs429358 in both schizophrenia and controls were consistent with HWE (case: *p* = 0.52; control: *p* = 1.0; all: *p* = 0.82). Both *ApoE* rs429358 allele and genotype distributions between patients and healthy controls did not differ (both *p* > 0.05).

**Table 2:** Comparison of rs429358 allele and genotype frequencies between schizophrenia cases and controls

Models	Genotypic	Cases	Controls	χ^2^	OR (95%CI)	*p* vaule	*p* adjusted*
Allele							
	C (n%)	77 (8.0)	69 (8.0)	0.218	0.92 (0.66–1.29)	0.64	0.71
	T (n%)	935 (92.0)	773 (92.0)		1.00		
Codominant							
	CC (n%)	4 (0.8)	2 (0.5)	0.922	1.64 (0.30–8.98)	0.63	0.12
	CT (n%)	69 (13.6)	65 (15.4)		0.87 (0.60–1.25)		
	TT (n%)	433 (85.6)	354 (84.1)		1.00		
Dominant							
	TT (n%)	433 (85.6)	354 (84.1)	0.396	1.00	0.53	0.45
	CC + CT (n%)	73 (14.4)	67 (15.9)		0.89 (0.62–1.28)		
Recessive							
	TT + CT (n%)	502 (99.2)	419 (99.5)	0.348	1.00	0.55	0.08
	CC (n%)	4 (0.8)	2 (0.5)		1.67 (0.30–9.16)		
Over-dominant							
	TT + CC (n%)	437 (86.4)	356 (84.6)	0.603	1.00	0.44	0.25
	CT (n%)	69 (13.6)	65 (15.4)		0.87 (0.60–1.25)		

^*^Adjusted by sex, age, and BMI

### Relationship between the ApoE rs429358 and clinical variables in patients

Table 3 shows the demographic data, clinical variables, and PANSS scores in the *ApoE* rs429358 genotype subgroups. There was a difference in BMI between the *ApoE* rs429358 genotype subgroups (*p* = 0.034). In addition, after controlling for the BMI, there was a trend toward significant difference in PANSS positive symptom score (*p* = 0.073), PANSS negative symptom score (*p* = 0.097), and PANSS total score (*p* = 0.087) between the *ApoE* rs429358 genotype subgroups.

**Table 3:** Demographic and clinical information according to *ApoE* rs429358 genotype of cases and controls in a Chinese sample

Variables	Cases (n = 506)					Health Controls (n = 421)			
	CT + CC (n = 73)	TT (n = 433)	t /X^2^	*p* value	*p* adjusted*	CT + CC (n = 67)	TT (n = 354)	t/X^2^	*p* value
Age (years)	47.45 ± 10.04	47.36 ± 9.66	− 0.077	0.939	–	46.15 ± 13.14	46.16 ± 13.27	0.006	0.995
Sex									
Female (%)	19 (26.0)	91 (21.0)	0.922	0.337	–	38 (56.7)	210 (59.3)	0.158	0.691
Male (%)	54(74.0)	342(79.0)				29 (43.3)	144 (40.7)		
Years of eudcation	10.21 ± 10.91	8.72 ± 2.60	− 1.159	0.250	–	10.80 ± 11.52	9.14 ± 3.32	-1.164	0.248
Body mass index	23.55 ± 4.12	24.73 ± 3.85	2.131	0.034	–	25.00 ± 3.58	25.41 ± 4.20	0.734	0.464
Age of onset (years)	23.34 ± 5.58	22.98 ± 4.77	− 0.587	0.558	–	–	–	–	–
Duration of illness (years)	24.11 ± 9.56	24.53 ± 10.07	0.335	0.738	–	–	–	–	–
Mean daily dose (mg/day) (chlorpromazine equivalents)	411.30 ± 180.56	386.20 ± 167.21	− 1.012	0.312	–	–	–	–	–
PANSS score									
Positive symptoms	12.34 ± 5.19	11.52 ± 4.71	− 1.357	0.175	0.073	–	–	–	–
Negative symptoms	25.16 ± 8.63	23.45 ± 8.36	− 1.608	0.109	0.097	–	–	–	–
General psychopathology	26.32 ± 6.09	25.47 ± 5.92	− 1.117	0.264	0.777	–	–	–	–
Total score	63.82 ± 14.51	60.45 ± 14.39	− 1.849	0.065	0.087	–	–	–	–
ApoA1 level ( g/L)	1.38 ± 0.40	1.52 ± 0.37	2.586	0.010	–	–	–	–	–
ApoB level ( g/L)	0.86 ± 0.26	0.88 ± 0.26	0.606	0.545	–	–	–	–	–
ApoA1/ApoB	1.71 ± 0.59	1.84 ± 0.64	1.448	0.148	–	–	–	–	–

^*^Adjusted by BMI

### Genotype effects on serum ApoA1 and ApoB levels between patients

ApoA1 levels were available for 507 patients, while ApoB levels were available for 505 patients. There were no ApoA1 and ApoB levels available for healthy controls. There was a significant difference in serum ApoA1 levels between the *ApoE* rs429358 genotype groups, showing that patients with TT genotype had higher ApoA1 levels than patients with CT + CC genotype (*p* = 0.010). After adjusting for BMI, the difference was still significant (*p* = 0.005). In addition, there was no significant difference in ApoB levels between the *ApoE* rs429358 genotype groups (*p* = 0.545).

### The effect of ApoE rs429358 on the relationship between ApoA1 levels and PANSS scores in patients

**Fig. 3:**
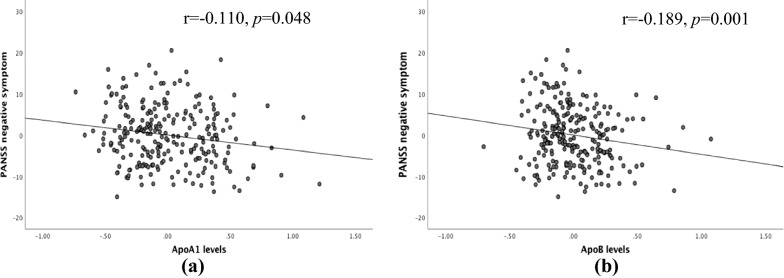
The partial correlation between ApoA1 (gl/L) and ApoB levels (gl/L) and PANSS scores of patients with schizophrenia. **a** ApoA1 levels and PANSS negative symptoms; **b** ApoB levels and PANSS negative symptoms

Pearson’s correlation analysis indicated that ApoA1 levels were positively correlated with PANSS positive symptom score (*r* = 0.142, *n* = 494, *p* = 0.001, pcor = 0.004) and PANSS general psychopathology score (*r* = 0.152, *n* = 494, *p* = 0.001, pcor = 0.004), while ApoA1 level was negatively correlated with the PANSS negative symptom score (*r* = − 0.160, *n* = 494, *p* < 0.001, pcor < 0.004). After adjusting for age, sex, BMI, years of education, and age of onset, only a significant partial correlation was identified between the ApoA1 levels and the PANSS negative symptom score (*r* = − 0.110, *n* = 323, *p* = 0.048, pcor = 0.192, Fig. 3). Furthermore, linear regression analysis identified that ApoA1 level was a predictor of PANSS negative symptom score (*t* = − 1.988, *p* = 0.048, pcor = 0.192).

Additionally, only in the T homozygote group, ApoA1 levels were positively correlated PANSS positive symptom score (*r* = 0.203, *n* = 335, *p* < 0.001, pcor < 0.004) and PANSS general psychopathology score (*r* = 0.219, *n* = 335, *p* < 0.001, pcor < 0.004), but negatively correlated with the PANSS negative symptom score (*r* = − 0.165, *n* = 335, *p* = 0.002, pcor = 0.008). Partial correlation analysis revealed that adjusting for age, gender, BMI, years of education, and age of onset, only the PANSS negative symptom score was correlated with the ApoA1 level (*r* = − 0.130, *n* = 236, *p* = 0.045, pcor = 0.180). Moreover, linear regression analysis confirmed that ApoA1 level was a predictor of PANSS negative symptom score in patients with T homozygotes (*t* = − 2.694, *p* = 0.008, pcor = 0.032).

Only in the C allele carriers, there was a trend toward significant correlation between PANSS negative symptom score and ApoA1 levels (*p* = 0.082, pcor = 0.328). While controlling for age, gender, BMI, years of education, and age of onset, no correlation was observed between the PANSS negative symptom score and the ApoA1 levels (*p* = 0.127, pcor = 0.508), which was also confirmed by linear regression analysis (*t* = − 1.562, *p* = 0.127, pcor = 0.508).

### The effect of ApoE rs429358 on the relationship between ApoB levels and PANSS scores in patients

Pearson’s correlation showed that ApoB levels were negatively association with PANSS negative symptom score (*r* = − 0.231, *n* = 492, *p* < 0.001, pcor < 0.004) or PANSS total score (*r* = − 0.105, *n* = 492, *p* = 0.020, pcor = 0.080). After controlling for age, gender, BMI, years of education and age of onset, there was still a significant correlation between the PANSS negative symptom score and ApoB levels (*r* = − 0.189, *n* = 321, *p* = 0.001, pcor = 0.004, Fig. 3). Further linear regression analysis identified that ApoB level was a predictor of PANSS negative symptom score (*t* = − 3.447, *p* = 0.001, pcor = 0.004). In addition, only in the T homozygote group, a significant correlation was observed between the ApoB levels and PANSS negative symptom score in all patients (*r* = − 0.209, *n* = 334, *p* < 0.001, pcor < 0.004). Further linear regression analyses confirmed that ApoB level was a predictor of PANSS negative symptom score (*t* = − 2.529, *p* = 0.012, pcor = 0.048).

Partial correlation analysis revealed that after adjusting for age, gender, BMI, years of education, and age of onset, only the PANSS negative symptom score was correlated with ApoB levels (*r* = − 0.1307, *n* = 235, *p* = 0.036, pcor = 0.144). Only in the C allele carriers, there was a significant correlation between the PANSS negative symptom score and ApoB levels (*r* = − 0.274, *n* = 57, *p* = 0.039, pcor = 0.156) However, controlling for age, gender, BMI, years of education, and age of onset, no correlation was observed between the PANSS negative symptom score and ApoB levels (*p* = 0.072, pcor = 0.288), which was also confirmed by linear regression analysis (*t* = − 1.856, *p* = 0.072, pcor = 0.288).

## Discussion

To our best knowledge, this is the first report to explore the relationship between *ApoE* rs429358 and clinical symptoms of patients with schizophrenia. We did not find an association between *ApoE* rs429358 and susceptibility to schizophrenia or clinical symptoms of schizophrenia. However, we found a significant association between serum ApoA1 or serum ApoB levels and PANSS negative symptoms, which was regulated by *ApoE* rs429358.

### No association between ApoE rs429358 and susceptibility and clinical symptoms of schizophrenia

This study revealed that there was no association between *ApoE* rs429358 and schizophrenia, which was similar to some previous studies [57, 58]. However, a previous meta-analysis reported an association of ApoE ε3 with schizophrenia in an Asian population [20]. Another study also found that there was a highly significant association between *ApoE* genotype and schizophrenia in a Chinese population [59]. Moreover, a French association study and meta-analysis showed that only in a male sample, there was an association between schizophrenia and *ApoE* ε2ε3 genotype [60]. In addition, an association between undifferentiated type of schizophrenia and *ApoE* ε3/ε3 genotype was found in a Serbian population [61]. Both *ApoE* ε3 and *ApoE*-219G haplotypes increased the risk of schizophrenia in siblings [62]. By comparing the above studies, we speculate that these different results between our current study and other studies may be partly due to differences in population, sample composition, type of schizophrenia, and synergy/interaction effects with other variants. Also, the age of patients could be a potential reason, and a meta-analysis indicated that *ApoE*-ε4 may play an age-mediated pathophysiological role in schizophrenia [63].

In disagreement with previous studies, our study found no association between *ApoE* rs429358 and any PANSS scores. One previous study indicated that the phenotypes of drug-free schizophrenia patients with and without the *ApoE* ε4 allele were different, showing that *ApoE* ε4 + patients had lower positive symptoms as measured by the Brief Psychiatric Rating Scale compared to *ApoE* ε4- patients [58]. Another study found that the frequency of the ε4 allele was significantly higher in schizophrenia patients with positive symptoms [64]. A study also found that *ApoE* ε2 genotype was associated with more depressive symptoms in older Chinese schizophrenia patients[65]. One possible reason for this is that the combined effect of rs429358 and rs7412, but not rs429358 alone, has a significant effect on the PANSS score of patients with schizophrenia. Furthermore, a longitudinal study found that *ApoE*-ε4 predicted worsening severity of hallucinations and delusions in patients with schizophrenia in late adulthood [63]. Hence, it is worth to perform a follow-up study between *ApoE* rs429358 and subsequent PANSS scores.

### Serum ApoA1 levels, ApoB levels, and ApoE rs429358 genotype groups in patients

Our finding that ApoA1 levels were significantly different between *ApoE* rs429358 subgroups. However, our study only showed that ApoB levels in TT genotype carriers were higher than those in CT + CC carriers, but the differences were not significant between the *ApoE* rs429358 subgroups. These findings are not in agreement with a study that found significantly lower ApoA1 levels but higher ApoB levels in Alzheimer disease (AD) patients carrying *ApoE* ɛ4 allele [44]. Potential reasons included differences in disease (schizophrenia vs. AD) and different composition of variants (rs429358 alone vs. a combination of rs429358 and rs7412).

Numerous studies in the general population have found that ApoA1 levels and ApoB levels are different between *ApoE* rs429358 genotype subgroups. To demonstrate this, one study found that *ApoE* ε4 allele carriers had significantly higher levels of ApoB in a community-based older individuals [42], while another study found that *ApoE* ε2 allele carriers had significantly lower levels of ApoB in Chinese Han population [41]. Equally important, a UK Biobank study revealed that ApoA and ApoB levels were found to be significantly different for each *ApoE* genotype with reference to ε3ε3 [45]. The disease population (healthy persons vs. chronic schizophrenia patients) may be a major possible explanation. It is well known that some antipsychotic drugs (e.g., clozapine, olanzapine, etc.) may affect the body's metabolic levels [66, 67].

### Association between PANSS negative symptom score and ApoA1 and ApoB levels

Our study found that ApoA1 and ApoB levels were positively associated with PANSS negative symptoms in patients with schizophrenia. Similarly, a previous study showed that ApoA1 levels were negatively associated with psychopathology score (i.e., unusual thought content, grandiosity, elated mood, motor hyperactivity, and distractibility), while ApoB levels were positively associated with psychopathology (i.e., grandiosity, elated mood, motor hyperactivity) in male patients with psychosis [68]. In addition, there was a significant association between tardive dyskinesia (TD) status and ApoA1 and ApoB levels [69] as well as between cognitive function and ApoA1 and ApoB levels [70]. A possible hypothesis is that the improvement in psychopathology that accompanies changes in ApoA1 and ApoB levels may be a side effect indicating the efficacy of antipsychotic drugs, similar to the effect of antipsychotic-induced weight gain [51, 71]. A number of literatures have revealed that obesity increases the ApoB /ApoA1 ratio [72] and that ApoA1 levels were positively associated with the risk of metabolically healthy obesity (MHO), but the ApoB /ApoA1 ratio is negatively associated [73].

### Association between serum ApoA1 or ApoB levels and PANSS negative symptom score was regulated by ApoE rs429358

Our study found that *ApoE* rs429358 genotype groupings had a significant effect on the relationship between serum ApoA1 or ApoB levels and negative symptoms of schizophrenia. The correlation between serum ApoA1 or ApoB levels and negative symptoms was observed only in the T homozygote group but not in the C allele group, which may provide a clue to the unusual findings that higher ApoA1 or ApoB levels were associated with better PANSS negative symptoms. There is evidence that some neuropsychiatric symptoms (e.g., depression, anxiety, apathy, agitation, aggression, hallucinations, and delusions) in AD patients are associated with the *ApoE* ε4 allele [21–23]. Furthermore, in our study, given that TT genotype carriers had higher serum ApoA1 levels than CT + CC carriers, this indicates that the *ApoE* rs429358 polymorphism may regulate ApoA1 levels. Moreover, the regulatory effect of the rs429358 polymorphism on ApoB levels may be subtle but still effectively affects the relationship between ApoB levels and psychiatric symptoms. Thus, *ApoE* genotype may play a role in conferring negative symptoms in schizophrenia by affecting ApoA1 or ApoB levels. We speculate that since negative symptoms are associated with certain brain structures (e.g., right frontal cortical surface area) [74] and *ApoE* genotype appears to be associated with whole brain structures [75], *ApoE* genotype also may indirectly influence the above relationships by modulating brain structures. However, we still need to further explore the specific mechanisms by which ApoE rs429358 affects the association between ApoB levels and psychiatric symptoms.

This study had several limitations. First, owing to the cross-sectional study design, the causality between *ApoE* rs429358, ApoA1 and ApoB levels, clinical symptoms in patients with schizophrenia was not directly presented. Second, the inpatients in this study had more severe psychopathology, longer duration of disease, and antipsychotic treatment than typical psychiatric outpatients or first episode and drug-naive patients with schizophrenia, which may limit the generalization of our findings in this study. Third, we only examined a single genetic polymorphism effect in this study, and we need to detect other functional variants of the *ApoE* gene, especially rs7412, because other polymorphisms, haplotypes, genes interaction, or gene–environment interaction may be associated with schizophrenia or with psychopathological symptoms of schizophrenia. Fourth, some potential covariates that may influence the blood lipid profile, such as dietary habits, physical exercise, and relevant treatments were not collected in the study. Fifth, we assessed serum apolipoprotein levels only in the case group, but not in the control group. Therefore, the observed relation between *ApoE* variant status and alterations in ApoA1 levels is specific for patients with schizophrenia. Sixth, the relatedly small sample size in this study may impact the generalization of the findings.

## Conclusions

In conclusion, *ApoE* rs429358 gene polymorphism did not directly affect the susceptibility to schizophrenia or psychiatric symptoms of schizophrenia. The association between serum ApoA1 and ApoB levels and clinical symptoms in patients with schizophrenia was regulated by the presence of *ApoE* rs429358 polymorphism. Nevertheless, due to the limited sample size and relatively low statistical power, our findings are still preliminary. Therefore, future studies are needed to confirm our current findings in larger samples from different ethnics, and the biological mechanisms of clinical symptoms of schizophrenia involved in *ApoE* rs429358 should be further studied.

## Data Availability

Data are available on reasonable request from the authors.

## Abbreviations

ApoE: Apolipoprotein E
ApoA1: Apolipoprotein A1
ApoB: Apolipoprotein B
PANSS: Positive and Negative Syndrome Scale
GDP: Gross domestic product
GBD: Global burden of disease
YLD: Years of life lived with disability
BDNF: Brain-derived neurotrophic factor
ALFA: Allele Frequency Aggregator
LDL: Low-density lipoproteins
HDL: High-density lipoproteins
VLDL: Very-low-density lipoproteins
AD: Alzheimer disease
SCID: Structured Clinical Interview for DSM-IV
ICC: Inter-rater correlation coefficient
MALDI-TOF–MS: Matrix-Assisted Laser Desorption/Ionization Time of Flight Mass Spectrometry
HWE: Hardy–Weinberg equilibrium
ANCOVA: Analysis of covariance
TD: Tardive dyskinesia
MHO: Metabolically Healthy Obese
